# STAT1-Induced Upregulation lncRNA LINC00958 Accelerates the Epithelial Ovarian Cancer Tumorigenesis by Regulating Wnt/*β*-Catenin Signaling

**DOI:** 10.1155/2021/1405045

**Published:** 2021-11-08

**Authors:** Min Xie, Qi Fu, Pin-pin Wang, Yu-lan Cui

**Affiliations:** Department of Gynecology and Obstetrics, The Second Affiliated Hospital of Harbin Medical University, Harbin, Heilongjiang, China

## Abstract

**Background:**

Growing studies have demonstrated that long noncoding RNAs (lncRNAs) play important roles in tumor progression. In this study, we aimed to explore the potential roles of lncRNA LINC00958 (LINC00958) and its biological functions in epithelial ovarian cancer (EOC).

**Methods:**

The expression of LINC00958 in 11 cases of EOC and adjacent nontumor specimens and five cell lines was detected by qRT-PCR. CCK-8, colony formation, and flow cytometry assays were conducted to study the cell viabilities of EOC cells. Wound scratch and transwell analyses were carried out for the examination of cell invasion and migration of EOC cells. The targeting associations between LINC00958 and STAT1 were demonstrated by ChIP analyses combined with luciferase reporter assays. The related proteins of Wnt/*β*-catenin signaling were determined using RT-PCR.

**Results:**

Higher levels of LINC00958 were observed in EOC tissues and cell lines. Our data also revealed that high LINC00958 expression was partly induced by STAT1. Functionally, knockdown of LINC00958 suppressed the proliferation, migration, and invasion of EOC cells. Mechanistic investigation showed that the inhibitory effect of LINC00958 knockdown on EOC cells was mediated by the Wnt/*β*-catenin signaling.

**Conclusion:**

Our findings suggested that STAT1-induced overexpression of LINC00958 promoted EOC progression by modulating Wnt/*β*-catenin signaling.

## 1. Introduction

Ovarian cancer is the 8th most common gynaecological cancer worldwide and regarded as one of the most frequent tumor-associated deaths in females, with a growing incidence in China [1, 2]. Epithelial ovarian cancer (EOC) is estimated to account for >80% of all cases of ovarian cancer. Of these, high-grade tumors are the most common and have an estimation of >50–60% of cases [3]. Although there are improvements in operative treatment and chemotherapy in EOC patients, more than 80% of the patients with advanced stages will undergo tumor recurrences and metastasis, leading to the median survival of 2–3 years [4, 5]. Thus, further insight into the potential mechanisms underlying recurrences and metastasis of EOC is needed urgently for the improvement of effective diagnostic and treatment targets for this tumor.

Growing biological researches involved in microarray analysis have shown that the estimation of 75–85% of human genomes is transcribed into noncoding RNAs [6]. Long noncoding RNAs (lncRNAs) are another class of noncoding RNA with >210 nucleotides in length and have limited abilities of coding normal proteins due to the lack of a complete open reading frame [7]. It has been confirmed by the use of *in vitro* and *in vivo* assays that lncRNAs exhibited their function of regulators in human progress by participating in various physiological and pathological processes [8, 9]. Given the critical roles of lncRNAs in the modulation of genes, especially tumor-associated genes, the possible associations between dysregulated lncRNAs and tumor progression attract growing attentions [10, 11]. Of note, some well-studied lncRNAs have been identified to be functional regulators via acting as antioncogenes, tumor promoters, or both, depending on the types of tumors [12, 13]. Until now, although many lncRNAs have been reported to be dysregulated in EOC, only a few lncRNAs have been functionally characterized [14]. In addition, it remains fully clear whether other lncRNAs are involved in the progression of EOC.

lncRNA LINC00958 (LINC00958), located on 11p15.3, was originally described to be a bladder cancer-related lncRNA by Seitz et al. [15]. Subsequently, several studies also reported that LINC00958 was abnormally expressed in other tumors, such as pancreatic cancer, gastric cancer, and glioma [16–18]. Some functional assays have confirmed LINC00958 as a tumor promoter in these cancers. However, the possible functions and underlying mechanism of LINC00958 in EOC remain to be explored. In this study, we firstly identified LINC00958 as an EOC-related lncRNA and reported its involvement in EOC progression.

## 2. Materials and Methods

### 2.1. Patients and Tissue Samples

All the tumor samples and matched normal samples were collected from EOC patients who were undergoing clinical operations. The study included 11 patients suffering from EOC. None of the patients received chemotherapy or radiotherapy prior to surgery. Written informed consent was obtained from all participants involved in the study, and this study was approved by the Ethical Committee of The Second Affiliated Hospital of Harbin Medical University.

### 2.2. Cell Culture and Transfection

The human EOC cell line (SKOV3, A2780, CAOV3, and OVCAR-3) was obtained from ATCC (Manassas, VA, USA). Cells were cultured in RMPI-1640 medium (#22314, Jiman Biotechnology, Pudong, Shanghai, China) supplemented with 10% FBS (Gibco, Shijingshan, Beijing, China), 100 U/mL penicillin (#61-33-6, Yuzhimei, Zhengzhou, Henan, China), and 100 *μ*g/mL streptomycin (Yuzhimei, Zhengzhou, Henan, China). EOC cells and normal cells were maintained at 37°C in a humidified atmosphere with 5% CO_2_.

For suppression of LINC00958, CAOV3 and SKOV3 were transfected using si-Lnc-1 (siRNA1 targeting LINC00958) and si-Lnc-2 (siRNA1 targeting LINC00958) which were synthetized (GEMA Gene Company, Pudong, Shanghai, China) and then selected with puromycin (Yuzhimei, Zhengzhou, Henan, China) for two weeks. The same cells without treatments were used as the control. Transient transfections were carried out by the use of the Lipofectamine 2000 (Invitrogen) following instructions of users. When the time of transfection exceed 48 h, the cells were saved for subsequent cell assays.

### 2.3. RNA Isolation and qRT-PCR

Total RNAs from EOC cells and tissue specimens were isolated using TRIzol reagent (Invitrogen, USA). The collected RNAs were reverse transcribed into cDNAs using the PrimeScript RT-PCR Kit (TaKaRa, Haidian, Beijing, China). The levels of lncRNA and related genes were examined by the use of qRT-PCR methods by applying SYBR Green qPCR Master Mix (TaKaRa, Haidian, Beijing, China). The experiment progresses were operated using the ABI 7500 Real-Time PCR System (Biosystems, USA). The reaction situations were as follows: predenaturation at 95°C for 12 min for one cycle, denaturation at 95°C for thirty seconds, and extensions at 72°C for thirty seconds. The primers used were 5′-GAACTCACCTACTCGCTCATC-3′ (forward primer) and 5′-CCGAAACTCATTCCCTTGGTTG-3′ (reverse primer) for LINC00958 and 5′-ATCAGGCTCAGTCGGGGAATA-3′ (forward primer) and 5′-TGGTCTCGTGTTCTCTGTTCT-3′ (reverse primer) for STAT1 and 5′-CTGGGCTACACTGAGCACC-3′ (forward primer) and 5′-AAGTGGTCGTTGAGGGCAATG-3′ (reverse primer) for GAPDH. Their specificity and efficiency were demonstrated. The GAPDH (housekeeping gene) was used as loading controls. The relative expression of lncRNAs was calculated using the 2^−ΔΔ*Ct*^ method.

### 2.4. Cell Proliferation Assays

Cell proliferation was explored using Cell Counting Kit-8 (Beyotime, China) for the exploration of functional influence of LINC00958. In brief, transfected cells were seeded into 96-well culture plates in 250 *μ*L/well and incubated at 37°C. Next, 15 *μ*L of CCK8 (thiazolyl blue) solution was added to each well and samples were incubated for 1 h at 37°C. A microtiter plate reader (PerkinElmer, Nanjing, Jiangsu, China) was used for the examination of the light absorbance of the solutions. For the colony formation assays, CAOV3 and SKOV3 cells were seeded in 6-well plates at a density of 800 cells per well and cultured for 14 days. Then, the PBS was used to wash the plates. Colonies were then fixed with 5% formaldehyde (Xuhui Technology, Nanjing, Jiangsu, China) for 15 minutes and stained for 15 minutes with 1.0% crystal violet. The visible colonies were manually counted.

### 2.5. Cell Migration Assay

The *in vitro* wound healing assays were used for the determination of roles of LINC00958 on cell migration. Briefly, SKOV3 and CAOV3 after transfection were first seeded in a six-well plate. After cells developed a monolayer that took over 95% of the surface field, they were rinsed in PBS twice for the elimination of floating cells. One night after inoculation, a scratch was created by the use of a 10 *μ*L pipette tip when disciple cells were noticed. A light microscope was used to photograph the fields between the gap of 0 and 48 h.

### 2.6. Cell Invasion Assays

After 48 h transfection, cells were resuspended into serum-free medium. 3 × 10^4^ cells were inoculated into the upper culture chamber coated with Matrigel. 600 *μ*L medium containing 10% FBS was placed in the lower chamber of transwell. After 24 h of incubation, the cells which were on the filter surface were further fixed with methanol and stained with crystal violet. Three random fields were selected and images were captured under a light microscope (Nikon, Pudong, Shanghai, China).

### 2.7. Apoptosis Assays

For the apoptosis assays, the Annexin V-FITC Apoptosis Kit (ab14033) was purchased from Gemini Technology (Pudong, Shanghai, China) and the procedures using standard experiment methodologies were as follows: cells were transfected as described above and were harvested and resuspended in binding buffer (Biomiga, Haidian, Beijing, China). 80 *μ*L of collected suspensions was added to FACS tubes and stained by the use of Annexin V-FITC and PI. Finally, the apoptosis rates of SKOV3 and CAOV3 cells were examined by applying a flow cytometer (Biosciences, Hangzhou, Zhejiang, China).

### 2.8. Luciferase Reporter Assays

The JASPAR procedures were conducted for the preliminary prediction of potential transcription factor binding sites at the promoter regions of LINC00958. Two binding motifs of LINC00958 were demonstrated. The different fragment sequences were synthesized by Genome Biology (Nanjing, Jiangsu, China) and then inserted into a pGL3-basic vector which was provided by doctor Lin (Hangzhou, Zhejiang, China). Then, the cotransfection using the above plasmids and pcDNA-STAT1 or negative controls were conducted. The successful integration for demonstration of sequences into the vector was confirmed using sequencing methods. Then, the luciferase activities were examined with the luciferase reporter systems.

### 2.9. Chromatin Immunoprecipitation (ChIP) Assays

The EZ-Magna ChIP Kit (EMD Millipore) was purchased and applied for the conduction of the ChIP assays following the product instructions. SKOV3 cells were treated with formaldehyde and incubated for 10 min to generate DNA-protein crosslinks. Then, the cells were lysed with cell lysis buffer (Kunming, Yunnan, China) and further handled using ultrasound to engender chromatin fragments of 250–400 bp. Antibodies including anti-STAT1 (Sino Biological, Haidian, Beijing, China) and IgG were applied for each immunoprecipitation. Finally, RT-PCR was performed to analyze the precipitated DNAs.

### 2.10. Statistical Analysis

All the statistical analyses were performed using SPSS13.0 for Windows (SPSS Inc., Chicago, IL, USA). Significance between two groups was assessed with Student's *t*-test. The differences were considered to be statistically significant at *p* < 0.05.

## 3. Results

### 3.1. LINC00958 Expression Is Increased in EOC Tissues and Cells

**Figure 1:**
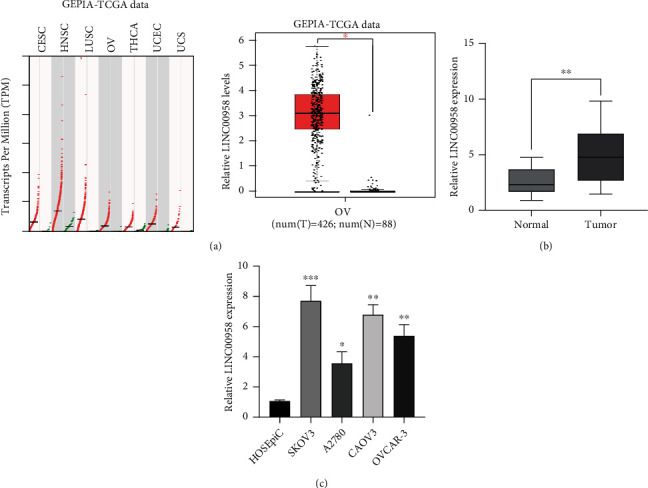
The expression levels of LINC00958 in EOC and its potential clinical value. (a) The expression pattern of LINC00958 in various tumor tissues was analyzed using “GEPIA.” (b) qRT-PCR was performed for the examination of the levels of LINC00958 in human EOC tissues and corresponding normal tissues. (c) The levels of LINC00958 in four EOC cell lines and normal HOSEpiC cells using RT-PCR. Assays were performed in triplicate. ^∗∗^*p* < 0.01 and ^∗^*p* < 0.05.

To explore whether LINC00958 was abnormally expressed in EOC, we analyzed RNA-Seq data from TCGA using an online procedure, GEPIA. As shown in Figure 1(a), a distinctly higher expression of LINC00958 was observed in EOC tissues compared to normal tissues. In order to demonstrate the above findings, we performed RT-PCR in EOC tissues and normal tissues from 11 EOC patients in our hospital: the results showed that LINC00958 levels in EOC tissues were distinctly higher than those in normal specimens (*p* < 0.01, Figure 1(b)). We further determined the LINC00958 levels in four EOC cell lines, SKOV3, A2780, CAOV3, and OVCAR-3, as well as in a normal mammary epithelial cell HOSEpiC (Figure 1(c)). Given a high level of LINC00958 in SKOV3 and CAOV3, we used the above two cells for further in vitro assays. Our results indicated that LINC00958 levels were distinctly upregulated in EOC cell lines. Overall, all the above results suggested LINC00958 as an overexpressed lncRNA in EOC.

### 3.2. Knockdown of LINC00958 Inhibited EOC Cell Proliferation and Promoted Apoptosis

**Figure 2:**
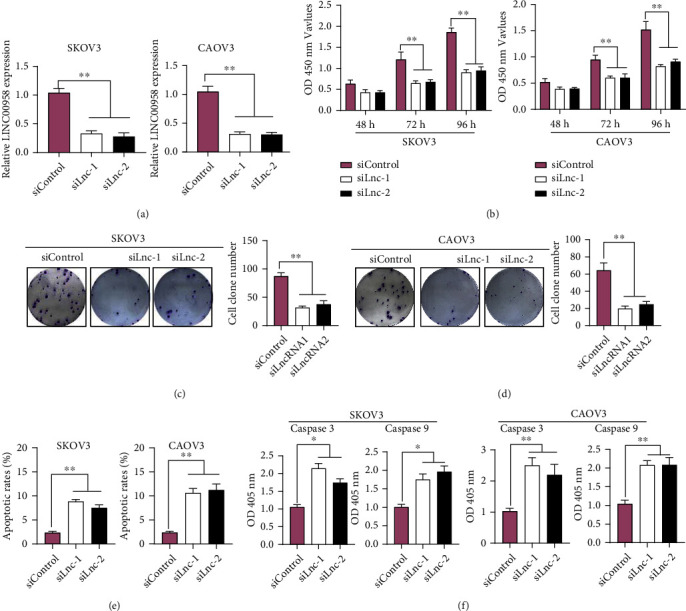
Downregulation of LINC00958 suppressed EOC cell proliferation and promoted apoptosis. (a) Knockdown of LINC00958 transfected with siLnc-1/2 in SKOV3 and CAOV3 cell lines. (b) SKOV3 cell viability and CAOV3 cell viability were determined using CCK-8 assays. (c, d) Colony formation assays. The potential effect of LINC00958 on SKOV3 and CAOV3 cell line colony formation abilities (magnification: 10x). (e) Cell apoptosis was examined by the use of Annexin V staining followed by flow cytometry. (f) The detection of Caspase 3/9 activity. ^∗∗^*p* < 0.01 and ^∗^*p* < 0.05.

Having confirmed LINC00958 is highly expressed in EOC. Our group then investigated its proproliferative functions on EOC cells. Using si-LINC00958 (siLnc-1/2), the interference efficiencies for decreasing LINC00958 expression are presented in Figure 2(a), which indicated that siLnc-1/2 was efficient to reduce LINC00958 expression. Function assays by the use of CCK-8 experiments suggested that LINC00958 knockdown suppressed cell proliferation in SKOV3 and CAOV3 cells (Figure 2(b)). Furthermore, the results of colony formation assays also suggested that silencing of LINC00958 distinctly repressed the proliferative abilities of SKOV3 and CAOV3 cells (Figures 2(c) and 2(d)). In addition, whether LINC00958 influenced the abilities of apoptosis was further explored. As shown in Figure 2(e), the percentage of apoptotic cells was distinctly increased with knockdown of LINC00958. To explore the potential mechanism involved in LINC00958-mediated apoptosis, we performed Caspase 3/9 activity assay, finding that downregulation of LINC00958 promoted the expression of Caspase 3/9 in SKOV3 and CAOV3 cells (Figure 2(f)). These results indicated that LINC00958 influenced the apoptotic functions by regulating Caspase 3/9. Overall, our results reminded LINC00958 as a tumor promoter in EOC cells.

### 3.3. Silence of LINC00958 Inhibits Migration and Invasion of EOC Cells

**Figure 3:**
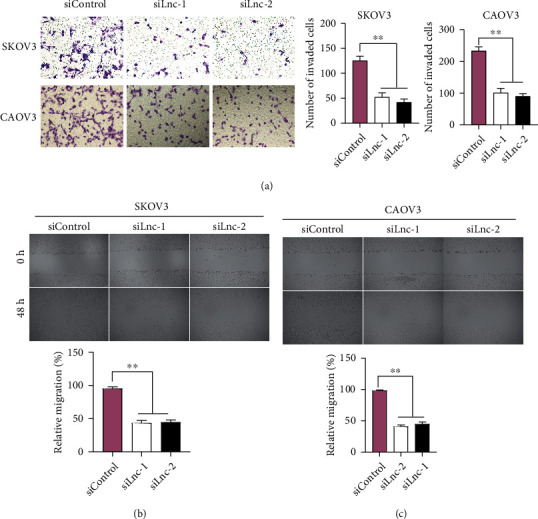
Downregulation of LINC00958 suppressed EOC cell migration and invasion. (a) Transwell invasion assays in LINC00958 stably depleted and control SKOV3 and CAOV3 cells (magnification: 40x). (b, c) Wound healing assays were conducted for investigation of changes in cell migration (magnification: 10x). ^∗∗^*p* < 0.01.

Metastasis, the transfer of disease from one organ or part to another, is the major cause of tumor-related deaths from tumors and occurred by the time of diagnosis in most patients [19]. The potential mechanism involved in the metastasis of tumor cells remained largely unclear. To explore whether LINC00958 displayed functional significance in the metastasis of EOC cells, we performed wound healing assays and invasion assays. As shown in Figure 3(a), the data showed that knockdown of LINC00958 suppressed the invasion abilities of SKOV3 and CAOV3 cells. The result of migration assays suggested that cell migration of SKOV3 and CAOV3 cells was distinctly suppressed by downregulating LINC00958 (Figures 3(b) and 3(c)). Overall, these results highlighted LINC00958 as an oncogenic lncRNA in the metastasis of EOC cells.

### 3.4. LINC00958 Upregulated by Transcription Factor STAT1

**Figure 4:**
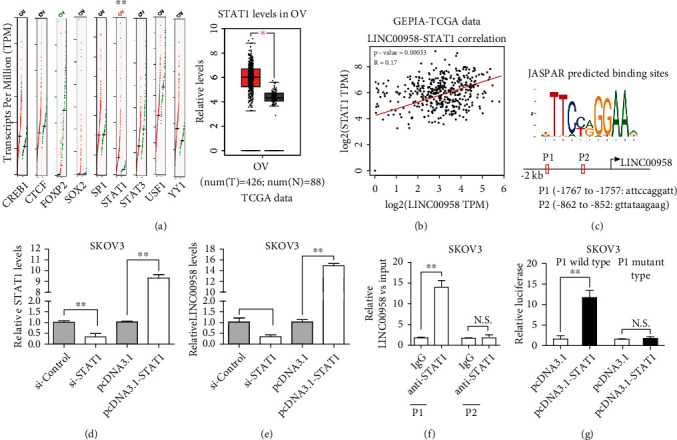
STAT1 is bound to the promoter region of LINC00958 and activated its transcription. (a) “GEPIA” showed the expression trend of several transcription factors. (b) The positive associations between LINC00958 and STAT1 levels. (c) JASPAR was used for the determination of STAT1 binding site prediction in the LINC00958 promoter region. (d) Expression levels of LINC00958 in SKOV3 cells after knockdown of STAT1 or overexpression of STAT1. (e) The levels of LINC00958 in SKOV3 cells after transfection. (f) ChIP assays were conducted to confirm the interaction between STAT1 and the LINC00958 promoter. (g) Dual-luciferase reporter assays were conducted to demonstrate the potential influence of overexpressed STAT1 on the luciferase activities. ^∗∗^*p* < 0.01 and ^∗^*p* < 0.05.

It has been confirmed that transcription factors were involved in the modulation of lncRNAs [20]. Then, we used the bioinformation software, “GEPIA,” to preliminarily screen potential transcription factors targeting LINC00958. As shown in Figure 4(a), a series of genes was identified to be highly expressed in EOC and our attention focused on STAT1. Then, the positive associations between LINC00958 levels and STAT1 levels were also confirmed using GEPIA (Figure 4(b)). Subsequently, the JASPAR CORE database was conducted for the analysis of the promoter of LINC00958. As shown in Figure 4(c), our group obtained the DNA motif of STAT1 and two binding sites of STAT1 were predicted in the LINC00958 promoter. To demonstrate previous results, we decreased or increased expression of STAT1 using si-STAT1 or pcDNA3.1-STAT1 in SKOV3 cells and the transfection efficiency was confirmed by RT-PCR (Figure 4(d)). Of note, knockdown of STAT1 distinctly suppressed the levels of LINC00958 in SKOV3 cells, while its overexpression displays an opposite trend (Figure 4(e)). The ChIP assay revealed that overexpression of STAT1 resulted in the occupancy of LINC00958 locus, suggesting the binding of STAT1 with the promoter region of LINC00958 (Figure 4(f)). Moreover, the results of luciferase studies also suggested that STAT1 induced the promoter activity of LINC00958 (Figure 4(g)). Taken together, our results indicated that upregulation of LINC00958 was induced by STAT1.

### 3.5. Knockdown of LINC00958 Represses Wnt/*β*-Catenin Signaling

**Figure 5:**
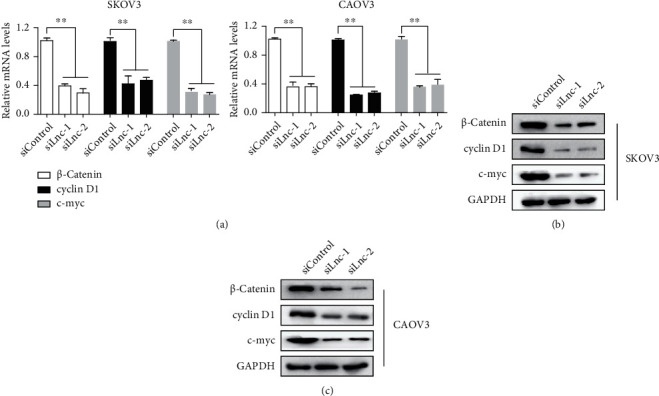
LINC00958 inhibits the Wnt/*β*-catenin signaling pathway. The mRNA (a) and protein (b, c) levels of *β*-catenin, cyclin D1, and c-myc in SKOV3 and CAOV3 cells transfected siLnc-1/2 were examined by RT-PCR and Western blot. ∗∗*p* <0.01.

Previous studies have demonstrated that Wnt/*β*-catenin signaling can cause uncontrolled cell growth and cell metastasis [21]. So, our group further delved into whether abnormally expressed LINC00958 altered the activity of Wnt/*β*-catenin signaling in EOC cells. The results of RT-PCR and Western blot indicated that the expression of *β*-catenin, cyclin D1, and c-myc in both mRNA and protein levels was distinctly inhibited after transfection with si-LINC00958 compared with that after transfection with siControl (Figures 5(a)–5(c)). Our findings confirmed LINC00958 as a regulator in the progress of Wnt/*β*-catenin signaling.

## 4. Discussion

EOC is a very complex disease with an unclear pathogenesis. The emerging significances of lncRNAs in cancers have become a hot spot. Previous evidences have revealed that lncRNAs are involved in the modulation of tumor growth and metastasis via the regulation of tumor suppressor or oncogenic pathways [22]. Recently, the positive associations between several lncRNAs and EOC have been reported. For instance, lncRNA SNHG20, an abnormally expressed lncRNA in several tumors, was reported to promote cell proliferation and metastasis, thus displaying oncogenic functions in EOC cells [23]. lncRNA LINC00460, a tumor-promoting regulator in EOC, was found to promote the growth and migration of EOC cells by modulating miR-338-3p [24]. These findings suggested that further exploration of potential functions of lncRNAs may provide novel insights that could help the improvement of early diagnosis, effective therapies, and prognosis prediction.

In this study, we identified a novel lncRNA LINC00958 involved in the progression of EOC. For the first time, we provided reliable evidences that LINC00958 expression was upregulated in EOC by performing bioinformatics analysis and RT-PCR in EOC tissues and cell lines. In addition, functional assays showed that silencing LINC00958 expression resulted in a distinct suppression of cell proliferation and metastasis and acceleration of EOC cell apoptosis. These results were in line with the findings from clinical assays that LINC00958 was associated with positively distant metastasis. Previously, several studies also reported that LINC00958 acted as a tumor promoter in several tumors. Our findings provided more evidences showing the cancer-promotive effects of LINC00958 on EOC.

Up to date, although the frequent dysregulation of lncRNAs has been confirmed in various tumors using a series of methods, the potential mechanism underlying the expressing differences of lncRNAs remains largely unclear. Emerging evidences showed that several transcription factors may be involved in the activation of lncRNAs. For instance, Zhao and Li [25] showed that overexpression of lncRNA HCP5 was induced by SP1. lncRNA HOXA10-AS expression was reported to be upregulated in lung adenocarcinoma, and this upregulation was induced by ELK1 overexpression [26]. In order to explore the mechanism involved in overexpression of LINC00958 in EOC, we applied the JARSPAR databases for the preliminary exploration of possible transcription factors binding with the promoters of LINC00958, finding out that STAT1 may be a candidate. Then, the results of luciferase assays and ChIP assays confirmed that LINC00958 was a transcriptional target of STAT1. The above essential date indicated that STAT1 activated LINC00958 translational expression in EOC.

Wnt/*β*-catenin signaling, a highly conserved pathway through evolutions, acts as an important regulator influencing embryonic development, cell polarity, differentiation, and stem cell renewal [27]. Recent findings revealed the critical effects of Wnt/*β*-catenin signaling in the modulation of immunomodulation, which highlighted its potential controls in pathogenesis of several cancers. In addition, Wnt/*β*-catenin activation was positively associated with the metastatic spread of various tumors, including EOC [28, 29]. Recently, growing studies reported the strong relationships between lncRNAs and Wnt/*β*-catenin signaling. Several functional lncRNAs have been shown to display tumor-controlling roles by modulating Wnt/*β*-catenin signaling [30, 31]. In this study, our group made an attempt to explore whether dysregulated LINC00958 influenced the activity of the Wnt/*β*-catenin pathway. The results of Western blot and RT-PCR showed that LINC00958 knockdown repressed *β*-catenin, cyclin D1, and c-myc expression in both mRNA and protein levels, which clearly revealed that the activity of the Wnt/*β*-catenin pathway was suppressed. Thus, these findings indicated that LINC00958 may show its oncogenic functions by modulating the Wnt/*β*-catenin pathway.

## 5. Conclusion

We firstly reported that STAT1-induced upregulation of LINC00958 promoted EOC cell proliferation and metastasis by epigenetically modulating the Wnt/*β*-catenin pathway. The important prognostic value of LINC00958 in EOC patients was also confirmed. LINC00958 could be a potential therapeutic target and prognostic biomarker for EOC patients. Further researches should be conducted for the study of the detailed mechanism involved in LINC00958 function.

## Data Availability

The data used to support the findings of this study are available from the corresponding author upon request.
